# Unravelling the function of funerary pottery vessels of the 2^nd^-1^st^ millennia BC in the Dailaman Province (Iran) through typology, petrography, and organic residue analyses

**DOI:** 10.1371/journal.pone.0306647

**Published:** 2024-09-16

**Authors:** Emmanuelle Casanova, Takehiro Miki, Yoshiki Miyata, Yoshihiro Nishiaki

**Affiliations:** 1 Laboratory of Climate and Environmental Sciences, UMR8212 CEA/CNRS/UVSQ, Gif-sur-Yvette, France; 2 The University Museum, University of Tokyo, Tokyo, Japan; 3 Archaeozoology and Archaeobotany: Societies, practices, Environment, UMR7209 Museum National d’Histoire Naturelle de Paris/CNRS, Paris, France; 4 Faculty of letters, Keio University, Tokyo, Japan; Istanbul University: Istanbul Universitesi, TÜRKIYE

## Abstract

Pottery vessels often comprise major burial goods at archaeological sites, thus providing valuable information for reconstructing past mortuary practices. However, because of the uncertainty of its function or use, which has been interpreted mostly through typological studies alone, the analytical potential of pottery as a burial good has not been fully exploited. This study applied bio-chemical and geochemical analyses for the first time to funerary pottery vessels of the Iron Age of North Iran to examine their function and use. The study materials are from the necropolis of Ghalekuti, Dailaman, excavated in the 1960s. Direct radiocarbon dating conducted on human and animal bones in the graves and typological analysis of the pottery anchored the chronological position of the pottery materials to the 2nd and 1st millennium BC. A petrographic analysis revealed that pottery vessels can be classified into six fabric types, including those with coarse tempers that are effective for cooking. Pottery pastes with finer inclusions less suited for cooking appeared during the early first millennium BC (Iron Age III). To obtain further insight into the function of the pottery, we conducted organic residue analyses. The results demonstrated that the vessels retained remains of botanical and animal origin. In particular, jars with tubular spouts, characteristic of the Iron Age III period, were likely specialised for botanical products. Interestingly, both carcass and dairy products from ruminant animals (cattle and caprine) were processed in short-neck jars and bowls, including spouted bowls, suggesting their use in a liquid state. Products from ruminants, particularly dairy products, may have played a significant role in the daily and ritual use of pottery vessels during the study period in Northern Iran. These results indicate that a range of pottery vessels used for specific purposes before the burial was offered for graves, helping us better understand the mortuary practices of Iron Age Iran.

## 1- Introduction

Pottery vessels are one of the most common archaeological finds. Therefore, they serve as a valuable source of information for understanding the cultural patterns of the human past. They provide clues not only about chronology and culture traditions but also the exploration of other diversified aspects of prehistoric society, including social systems, economies, diets, rituals, and beyond [1, 2]. This study presents the application of organic residue analysis (ORA), which has been rapidly developing in recent years, to pottery vessels recovered in the context of mortuaries in the Dailaman Province of Iran. Combined with typological and petrographic data, this study provides new data on funerary rituals in Iron Age Iran from the perspective of pottery analysis.

The function of pottery vessels can be utilitarian, highly related to the storage, transport, and cooking of food, or non-utilitarian related to symbolism and ritual purposes [3]. In the funerary contexts, there is an unresolved question as to whether these ceramic vessels were utilitarian and used in the lifetime of the deceased, whether they were empty ornamental objects made for deposition, or if they were actively used in ritual ceremonies. One way to understand pottery function is to characterize mineral and vegetal inclusion and performance characteristics through thin-section petrography. For instance, an experimental study examined the difference in performance characteristics between pottery fabrics with mineral temper, plant temper, and without temper [4]. The results of this study showed that mineral-tempered pottery indicated a superior function in heating effectiveness that would be more suitable for cooking than plant-based temper. Chalcolithic cooking pots found in southern Iran also included coarse and angular mineral temper, supporting the above-mentioned study [5]. Thin-section analyses of ceramics also permit us to gain insight into clay composition, origin, and manufacturing.

Another way to gain insight into pottery function is to identify traces of past contents through organic residue analysis (ORA). This method is largely employed on ceramics from domestic contexts to reconstruct paleodiet, However, it is now increasingly applied on ceramic vessels from funerary contexts. For instance, ORA on baby bottles from child graves in central Europe was likely employed to feed the child with ruminant milk in their lifetime [6], or horse carcass offerings in Bronze Age cemeteries of Kazakhstan [7]. Therefore, ORA is a relevant technique for studying the function of ceramic vessels in funerary contexts.

Here, we focused on cemeteries from Ghalekuti I and II in the Dailaman Province of Iran [8–10]. The preservation of complete ceramic vessels in the graves provides an opportunity to study the relationship between the forms, manufacturing, and use of pottery vessels for specific products. Only a few graves contained animal bones deposited with the deceased. In one case inside a pottery vessel which could suggest goods offerings (see below). In most cases, animal remains come from outside the graves and thus may not be contemporaneous with the burial, limiting our understanding of animal offerings during funerary rituals during the Iron Age. Herein, we conducted thin-section petrography and lipid residue analysis to study the function of various pottery typologies recovered during the Iron Age period in the Dailaman Province (Iran).

## 2. Materials and methods

### 2.1 The site and materials from Ghalekuti I and II

**Fig 1:**
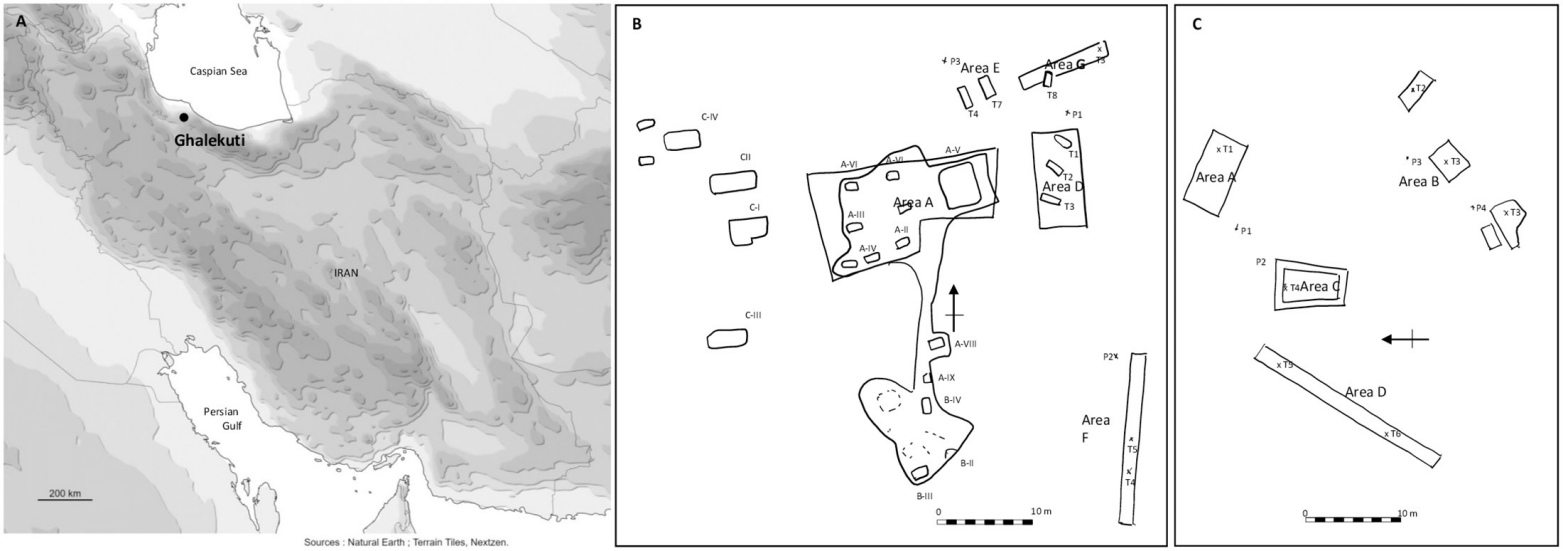
A- Map of Iran showing the location of Ghalekuti. B- Ghaleluti I excavated areas. C- Ghalekuti II excavated areas. Redrawn and adapted from Egami et al., (PL. XL) [8] and Fukai et Ikeda (PL. XLVI and XLII) [9].

The site of Ghalekuti corresponds to four hills in the Dailaman Province in the North of Iran (Fig 1A). Two of the mounds, Ghalekuti I and II, were excavated during the 3rd and 4th Tokyo University Iraq-Iran Archaeological expeditions directed by Namio Egami in 1960 and 1964. The archaeological prospections revealed large necropolises, consisting of at least 270 graves. A total of 26 non-looted graves were excavated at Ghalekuti I, and seven at Ghalekuti II (Fig 1B and 1C). The tombs were mainly divided into pit graves and stone chambers, some of which were reemployed in later periods. Associated with the burials, numerous objects such as pottery, bronze, iron, and glassware were discovered [8–10]. It offers one of the largest ceramic collections of 2^nd^ and 1^rst^ millennium BC from the Dailaman Province in Northern Iran. Materials and remains were divided between the National Museum of Iran, Tehran, Iran, and the University Museum, the University of Tokyo, Japan. Pre-Achaemenid Iron Age collections stored in Tokyo were the object of the current study.

The chronological interpretation of the tombs at Ghalekuti I and II deserves commentary. While the original excavation reports broadly assigned them to the Bronze to Iron Age, without detailed specification, Miyake’s [11] synthetic typological study identified three chronological groups: the Late Bronze Age, the Early Iron Age, and the Late Iron Age. This tripartite model was maintained by later studies, such as those from Haerinck [12] and Arimatsu [3], although the latter designated them as Iron Ages I, II, and III, respectively. The assignment of the oldest pottery assemblages to the Iron Age rather than the Bronze Age followed the then-prevalent notion that the production of ‘monochrome black ware’ is a hallmark of the Iron Age of the northern Iranian Zagros. However, the problems with the singular application of the term ‘Iron Age’ to this long chronological unit, which started in the mid-second millennium BC, far earlier than in the other parts of Southwest Asia, was obvious; a revised chronology has now been proposed for the Iron Age of the Iranian Zagros [13, 14]. Based on the solid stratified evidence obtained at Hasanlu, a key site in Northwest Iran, the Pre-Achaemenid Iron Age is defined as a period starting later and divided into Iron Age I (1250–1050 BC), II (1050–800 BC) and III (800–550 BC) [14, 15]. Given the wide acceptance of this revised chronology today [16], the chronology of the Ghalekuti materials should also be updated. In practice, this issue lies in the definition of Iron Age (hereafter IA) I ceramics. However, we have not assigned the pottery specimens in question to a specific period of Bronze or IA I because this task requires a comprehensive techno-typological study of pottery materials. Therefore, the related material for the present study was provisionally assigned to the Bronze Age/Iron Age I (BA/IA I). The materials described as IA II and III in this study fit well with the current chronological understanding [17].

**Fig 2:**
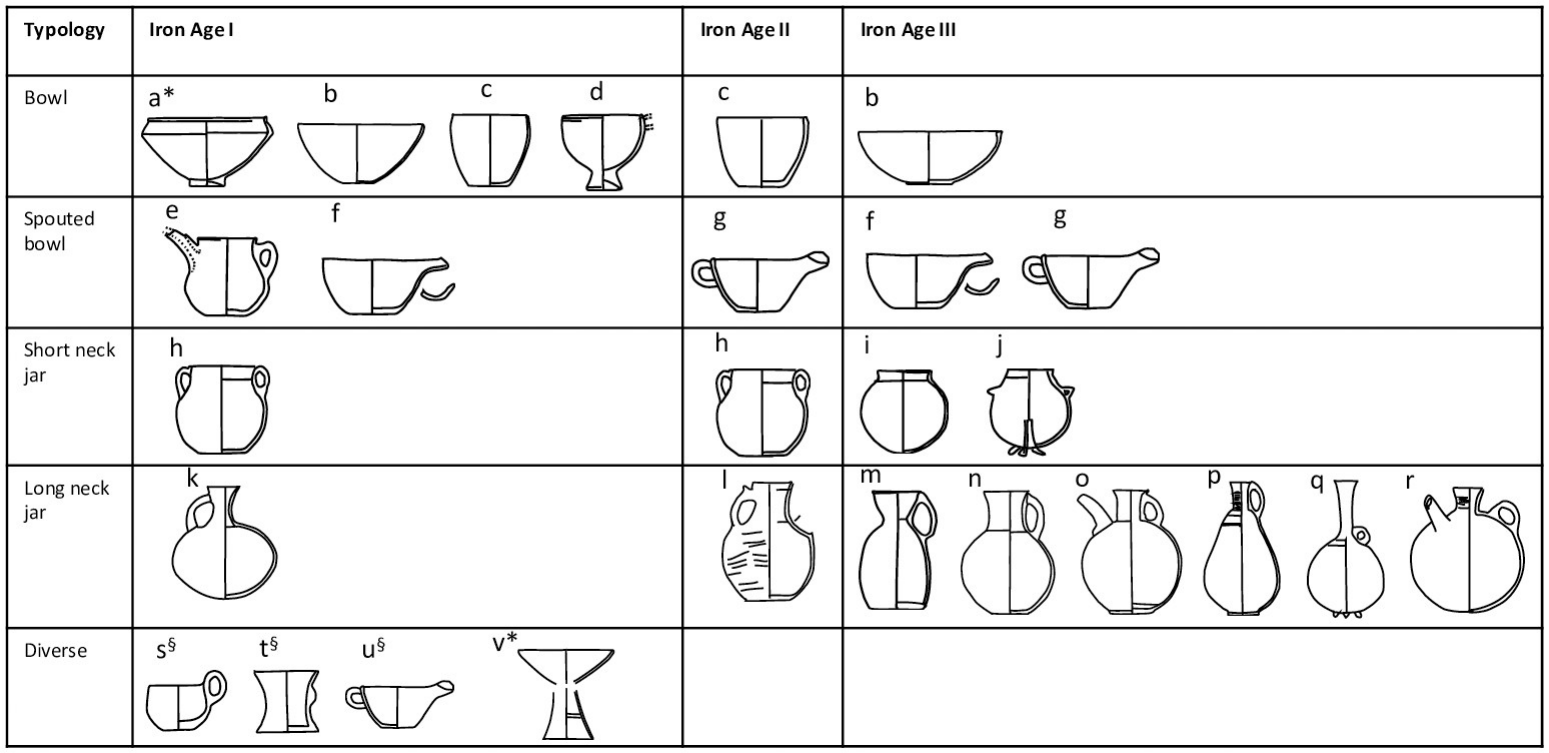
Drawings of the pottery typology studied for organic residue analyses (not scaled). Other typologies available at the site are not presented here. If several pottery vessels have the same letter it means it is the same typology, *denotes very large vessels, §denotes very small vessels. Redrawn and adapted from Egami et al. [8], Fukai and Ikeda [9] Sono and Fukai [3, 10].

The study materials comprise three major chronological groups (Fig 2). According to Arimatsu [3], assemblage A represents the oldest BA/IA I. It presents the highest diversity of forms that are dominated by large bowls and short neck jars, which are associated with bowls on foot, deep bowls, and large shallow bowls. In terms of surface colours, it mostly consists of dark-brown ceramic with coarse and fine specimens, as well as grey-brown and brown-red specimens. The IA II is represented by the assemblage B-II composed of plates, bowls with handles, bowls with spouts, small jars, and long neck jars. It is dominated by grey-brown pastes. The IA III period is represented by the assemblage B-III, composed of plates and small jars. It is dominated by fine red-brown ceramics and some coarse specimens of brown-red and grey-brown ceramics. Some of the forms are common in several assemblages, such as a bowl, long neck jar, and short neck jar, which testify to some level of continuity in vessel production across the Iron Age and Achaemenid period.

The present study is based on a collection housed at the University Museum, the University of Tokyo, in which more than 160 complete or nearly complete vessels and plenty of pottery fragments from Dailaman are available [18]. We sampled 40 specimens following two criteria. One is to give priority to the tombs that yielded more abundant complete or nearly complete pottery vessels, which have been given UMUT Registration # (Table 1) so that the pottery assemblage can be studied in the original form as much as possible. The other criterion is to consider the balance in typological as well as temporal distributions. Accordingly, for example, in the case of vessels with too narrow spouts or necks for their interior sampling, typologically comparable fragments were selected (7 pieces in Table 1). As a result, we studied an assemblage of 40 specimens from nine graves (A-III, A-IV, A-VI, A-VIII, B-III, B-IV, C-I, C-II, and G-9), consisting of 17 pieces from BA/IA I, four from IA II and 19 from IA III. The relatively poor representation of IA II reflects the chronological pattern of the grave compositions at Ghalekuti I and II [18].

**Table 1:** Pottery vessels studied for their lipid residue analysis, context, type (see Fig 2 for details), lipid concentration and source assignment.

POTIRAN#	Registration#	Grave	Phase	Type	Concentration (μg/g)	Lipid profile	δ^13^C_16:0_ (‰)	δ^13^C_18:0_ (‰)	Δ^13^C (‰)	Assignment
GHA-0541	3GHAI. P43	C-I	IA III	Long neck jar	10	2	-26,6	-27,4	-0,8	VO
GHA-0542	3GHAI. P44	C-I	IA III	Spouted bowl	52	1	-26,8	-29,2	-2,4	RAF
GHA-0543	3GHAI. P45	C-I	IA III	Long neck jar	32	2	-27,4	-26,6	0,8	VO
GHA-0544	3GHAI. P47	C-I	IA III	Short neck jar	-	-	-26,8	-26,7	0,1	*MC*
GHA-0545	3GHAI. P50	C-I	IA III	Long neck jar	8	2	-26,2	-27	-0,8	VO
GHA-0546	3GHAI. P52	C-I	IA III	Bowl	47	1	-30,2	-34,2	-4,0	RDF
GHA-0547	3GHAI. P55	C-I	IA III	Tubular spouted jar	-	-	-27,2	-26,4	0,8	*MC*
GHA-0548	3GHAI. P56	C-I	IA III	Diverse, spouted bowl	94	1	-28,9	-33,1	-4,2	RDF
GHA-0549	3GHAI. P57	C-I	IA III	Tubular spouted jar	11	2	-27,7	-27,9	-0,2	VO
GHA-0550	3GHAI. P58	C-I	IA III	Tubular spouted jar	112	2	-26,5	-26,6	-0,1	VO
GHA-0551	-	B III	IA III	Short neck jar	87	1	-28,8	-33,4	-4,6	RDF
GHA-0552	-	B III	IA III	Long neck jar	27	1	-24,6	-25,8	-1,3	VO
GHA-0553	4GHAII.P5	II-4	IA II	Short neck jar	137	1	-26,9	-30,8	-3,9	RDF
GHA-0554	4GHAII.P6	II-4	IA II	Spouted bowl	150	1	-28,2	-30,6	-2,4	RAF
GHA-0555	4GHAII.P7	II-4	IA II	Bowl	-	-	-27,9	-33,0	-5,1	*MC*
GHA-0556	4GHAII.P8	II-4	IA II	Tubular spouted jar	-	-	-26,6	-25,2	1,4	*MC*
GHA-0557	3GHAI.P8	A IV	BA/IA I	Short neck jar	306	1	-26,9	-31,2	-4,3	RDF
GHA-0558	3GHAI.P12	A IV	BA/IA I	Diverse, cup	247	1	-27,5	-32,3	-4,8	RDF
GHA-0559	3GHAI.P10	A IV	BA/IA I	Long neck jar	28	1	-26,2	-27,2	-1,0	RAF
GHA-0560	4GHAI.P13	G, 9	BA/IA I	Tubular spouted bowl	43	2	-27,1	-27,3	-0,2	VO
GHA-0561	4GHAI.P18	G, 9	BA/IA I	Bowl	440	1	-27,5	-29,9	-2,4	RAF
GHA-0562	4GHAI.P16	G, 9	BA/IA I	Bowl	111	1	-27,4	-31,2	-3,8	RDF
GHA-0563	3GHAI.P18	A VI	BA/IA I	Bowl	24	1	-28,4	-31,2	-2,8	RAF
GHA-0564	3GHAI.P22	A VIII	BA/IA I	Bowl on foot	33	1	-26,7	-29,7	-3,0	RAF
GHA-0565	3GHAI.P25	A VIII	BA/IA I	Large bowl	103	1	-26,8	-27,8	-1,0	RAF
GHA-0566	3GHAI.P60	C-I	BA/IA I	Bowl	75	1	-27,1	-31,6	-4,5	RDF
GHA-0567	3GHAI.P64	C II	BA/IA I	Large bowl on foot	101	1	-26,5	-28,8	-2,3	RAF
GHA-0568	3GHAI.P7	A III	BA/IA I	Large bowl	10	2	-28,0	-26,3	1,7	VO
GHA-0569	GHA 3 3p	A III	BA/IA I	Bowl	-	-	-27,0	-26,2	0,8	*MC*
GHA-0570	GHA 3 3p	A III	BA/IA I	Bowl on foot	30	1	-26,8	-27,6	-0,8	RAF
GHA-0571	3GHAI.P33	B III	BA/IA I	Short neck jar	5	1	-26,7	-28,6	-1,9	RAF
GHA-0572	3GHAI.P37	B III	BA/IA I	Diverse, cup	27	2	-27,4	-26,7	0,7	VO
GHA-0573	3GHAI.P42	C-I	IA III	Tubular spouted jar	27	2	-28,1	-26,2	1,9	VO
GHA-0574	3GHAI.P49	C-I	IA III	Tubular spouted jar	290	1	-29,3	-33,1	-3,8	RDF
GHA-0575	3GHAI.P54	C-I	IA III	Spouted bowl	33	1	-28,8	-32,5	-3,7	RDF
GHA-0576	3GHAI.P59	C-I	IA III	Long neck jar	44	2	-25,7	-26,3	-0,6	VO
GHA-0577	3GHAI.P62	C-I	IA III	Tubular spouted jar	77	2	-27,4	-30,1	-2,7	VO
GHA-0578	-	B III	IA III	Long neck jar	4	2	-26,8	-27,7	-0,9	VO
GHA-0579	-	B IV	IA III	Long neck jar	7	2	-27,4	-28,1	-0,7	VO
GHA-0580	-	C-I	BA/IA I	Spouted bowl	80	1	-27,2	-31,1	-3,9	RDF

IA = Iron Age, RAF = ruminant adipose fats, RDF = ruminant dairy fats, NRAF = non-ruminant adipose fats, VO = vegetable oil, MC = Modern contamination.

When dealing with the pottery materials from the Ghalekuti I and II necropolises, it is important to note that the same grave could have been used repeatedly in different periods. This possibility is applicable to our study graves, namely Tomb C-1, Tomb-9, and Tomb B-III. Tomb C-1 is one of the largest graves discovered at Ghalekuti, which clearly showed repeated use. The original grave was assigned to Layer IV, belonging to BA/IA I, overlain by later graves of IA III. Our study samples consist of both. Tomb-9 was also utilized in BA/IA I and IA III [8]. However, we sampled only pottery specimens from the BA/IA I grave associated with Skeleton 12. On the other hand, the situation of Tomb B-III is different. The excavators interpret that its upper layer (Layer I), which included stone features and occupational debris, could represent settlement remains [8] rather than grave goods. The grave itself was identified at the basal level (Layer I). Again, these two periods are assigned to IA III and BA/IA I respectively. The IA III samples from Tomb B-III contained no complete pottery, likely reflecting the different nature of their origin as suggested by the excavators.

### 2.2 Radiocarbon analyses

The chronology of the Dailaman necropolises has been discussed primarily on the basis of typological studies of pottery materials over more than a half-century [3]. In the present study, we carried out radiocarbon dating of archaeological materials that directly relate to our concern. The samples were chosen from the human and animal bones of Ghalekuti stored at the University Museum, the University of Tokyo. Considering the chronological distribution, three samples (one human and two animal bones) were chosen from the BA/IA I collection, whereas also three but all human remains for the IA III (Table 2). However, as there is no human bone available for the IA II [9, 10], we chose an animal bone supposedly from Tomb IV of Ghalekuti II belonging to this period. It is a scapula fragment of probably *Bos*. Most interestingly, the unpublished field archive mentions that it was “discovered in a pottery vessel”, although the details have not been published to date.

**Table 2:** Materials selected for radiocarbon dating, context, period, δ^13^C and δ^15^N values (recorded on an EA-IRMS), C/N ratios and conventional radiocarbon measurement and calibrated radiocarbon measurements in OxCal (v4.4) against IntCal20 calibration curve (Reimer et al., 2020).

UMT no.	Context	Period	Material	δ^13^C (‰)	δ^15^N (‰)	C/N	AMS Lab no.	^14^C Age BP	Calibrated age BC (68,3%)	Calibrated age BC (95,4%)
S-28282	GHA I-B3 (GhaB3X1)	IA III	human bone collagen	-19.2	7.5	3,3	TKA-28177	2507 ± 20	768–749 (12.1%) 686–666 (13.3%) 640–570 (42.8%)	775–732 (19.5%) 699–662 (18.3%) 651–544 (57.6%)
S-28283	GHA I-C1 (Gha17)	IA III	human bone collagen	-17.3	6.2	3,3	TKA-28178	2856 ± 21	1053–981 (63.7%) 948–939 (4.6%)	1112–968 (84.6%) 961–931 (10.9%)
S-28284	GHA I-T9 (Gh.S.09)	IA III	human bone collagen	-19.0	7.8	3,4	TKA-28179	2741 ± 20	906–892 (14.1%) 880–834 (54.2%)	926–826 (95.4%)
S-28285	GHA I-A4 (Gh.6)	BA/IA I	human bone collagen	-19.5	8.5	3,3	TKA-28180	3351 ± 22	1685–1652 (22.8%) 1644–1609 (31.1%) 1577–1561 (9.8%) 1554–1546 (4.5%)	1734–1719 (4.8%) 1690–1541 (90.7%)
S-28286	GHA I-A3	BA/IA I	animal bone (Bos) collagen	-20.2	6.3	3,3	TKA-28181	3307 ± 21	1611–1574 (40.7%) 1564–1536 (27.5%)	1620–1517 (95.4%)
S-28287	GHA I-C1	BA/IA I	animal bone (Ovis) collagen	-19.8	4.1	3,3	TKA-28182	3300 ± 21	1611–1574 (38.6%) 1564–1532 (29.6%)	1616–1513 (95.4%)
S-28288	GHA II-T4	IA II?	animal bone (probably Bos) collagen	-19.2	4.9	3,4	TKA-28183	2627 ± 20	807–795 (68.3%)	816–781 (95.4%)

The C/N, δ^13^C, δ^15^N, and radiocarbon analyses were conducted at the radiocarbon laboratory of the University Museum of the University of Tokyo. Collagen extraction was carried out following a modified Login protocol [19, 20]. In summary, bones were sonicated (ultra-pure water, 10min) and reduced to powder. The powdered bones were then demineralised (1.2M HCl, 4°C, 42h), washed (ultra-pure water, 4°C, 3.5h), cleared from humic acids (0.1M NaOH, 1.5h), washed (ultra-pure water, 4°C, 3.5h) and gelatinised (ultra-pure water, 90°C, 40.5h). Gelatine was filtered (Watman GF/F filters) and lyophilised. The C/N, δ^13^C, and δ^15^N of collagen were measured on a vario PYRO cube coupled to an Isoprime visION (Elementar). About 2.5mg of collagen was combusted on an Elemental Analyzer (Elementar, vario ISOTOPE SELECT), the CO_2_ was trapped into quartz tubes, then graphitized on iron catalyst (650°C, 6h). Radiocarbon measurements were calibrated using OxCal (v4.4.4) [21] against the IntCal20 calibration curve [22].

### 2.3 Thin section petrography analyses

A total of 12 potsherds from the BA/IA I (five samples) and IA III periods (seven samples), covering the range of clays available at the site, were selected for petrographic analyses. The full-shaped vessels were deemed unsuitable for these destructive analyses. The potsherds chosen for petrographic analyses were vertically sliced into approximately 0.5 cm sections using a rock cutting device, glued to microscope glass slides with epoxy resin, then cut to about 1 mm thickness, and manually polished to 0.03 mm thickness with a polishing glass plate and powder. Thin sections were classified based on their inclusions, clay matrix, and voids through observation under a polarising microscope (Meijitechno MT9200) using PPL and XP lighting [23, 24].

### 2.4 Lipid residue analyses

A total of 40 pottery vessels were studied for their lipid residue analyses. These correspond to the BA/IA I, II, and III assemblages (Fig 2). Many specimens underwent restoration after the excavation. Therefore, care was taken to sample the ceramic away from the restoration works. The specimens too heavily restored to offer the possibility to sample away from restoration areas were not considered in this study. For the vessels with a spout or a long neck, that suggests the pouring of commodities, sampling was performed directly in the spout or at the junction of the body to the spout to maximise the chances of finding proof of their use.

A small surface of the ceramic was removed with a modelling drill to get rid of surface contaminants. Then, about 100–150 mg of clay was sampled as a powder with another clean modelling drill and collected in aluminium foil before their analysis. Lipids were extracted from the sampled clay powder using a methanolic solution of sulphuric acid (2 mL, 10% v/v, 70°C, 4 h) followed by liquid-liquid extraction with n-hexane (3x 2 mL) to obtain the total lipid extract (TLE). The organic fraction was washed with anhydrous potassium carbonate and the excess solvent was dried under a gentle nitrogen stream before adding a standard (n-tetratriacontane, 10 μL). Extracts were treated with *N*,*O*-Bis (trimethylsilyl) trifluoroacetamide (20 μL, 70°C, 1 h) to derivatize potential *n*-alkanols present in the extract, before analyses by gas chromatography (GC), GC-mass spectrometry (GC-MS), and GC-combustion-isotope ratio MS (GC-C-IRMS).

Lipid quantification (on the palmitic and stearic acids only) was performed on a Shimadzu 2014 GC fitted with a DB-1HT column (15 m, 0.32 mm i.d., 0.1 μm film thickness) using the internal standard method. Molecule identification was performed on a TraceGC ultra coupled to an ISQ mass spectrometer fitted with an HP-1MS column (50 m, 0.32 mm i.d., 0.17 μm film thickness). Measures of δ^13^C_16:0_ and δ^13^C_18:0_ were performed on an Agilent7890B GC coupled to an Isoprime GC5 and Elementar Isoprime visION mass spectrometer fitted with an HP-1 column (60 m, 0.32 mm i.d., 0.25 μm film thickness). The δ^13^C values on individual fatty acids were measured in triplicates, and the average value was used for data processing.

## 3- Results

### 3.1 Radiocarbon dating of human and faunal remains

A total of seven radiocarbon measurements were performed on bone collagen from humans and animals recovered from the graves at Ghalekuti (Table 2). The C/N ratios suggest good collagen quality [25, 26], and the δ^13^C and δ^15^N appear coherent with a terrestrial diet (Table 2). Thus, a reservoir effect deriving from the consumption of aquatic resources by humans is not expected here.

**Fig 3:**
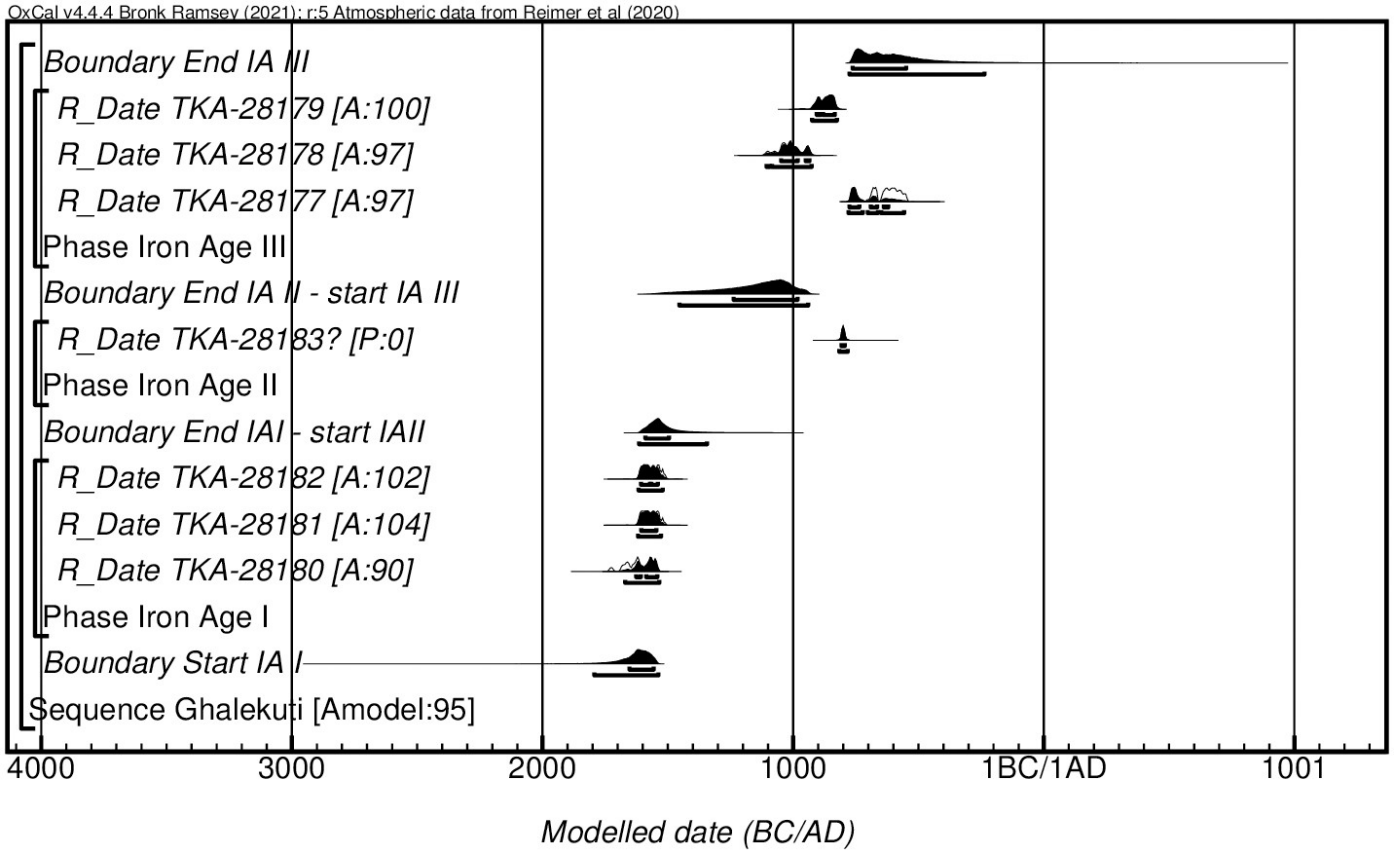
Probability distribution of radiocarbon measurement on human and animal bone collagen from Ghalekuti I and II. Each distribution represents the relative probability that an event occurs at a particular time. For each of the dates, two distributions have been plotted: one in outline, which is the result of simple radiocarbon calibration, and a solid one, based on the chronological model used. Distributions other than those relating to particular samples correspond to aspects of the model. The large square brackets down the left-hand side of the figure, along with the OxCal keywords, define the overall model exactly.

The ^14^C measurements have been calibrated in a sequence based on the succession of the BA/IA I, IA II, and IA III periods. In such a case, the model (not shown) has a poor agreement (A = 0.3), and two ^14^C dates are identified as outliers. When revising this model with only TKA-28183 measurement as an outlier in the sequence, the model reaches a good agreement (A = 98) (Fig 3). This suggests the single ^14^C date obtained for the IA II sequence is problematic and could more likely be of the IA III phase. It should, however, as mentioned earlier, be noted that only a single measurement was performed based on the scarce recovery of IA II material at the site, which is not enough to properly estimate the IA II occupation period at Ghalekuti.

The model suggests the duration of BA/IA I burials at Ghalekuti started in 1800–1538 cal BC (95.3%), probably in 1656–1556 cal BC (68.3%), and ended in 1615–1340 cal BC (95.4%), probably in 1591–1496 cal BC (68.3%). The model suggests the duration of IA III burials at Ghalekuti started in 1458–940 cal BC (95.4%), probably in 1237–985 cal BC (68.3%), and ended in 775–232 cal BC (95.4%), probably in 762–546 cal BC (68.3%). The three dates of the oldest group (TKA-28180, TAK-28181, and TAK28182) similarly indicate a date of the mid-2nd millennium BC, which confirms that the earliest graves at Ghalekuti were established in the Bronze Age. According to the chronology of Hasanlu, they correspond to the Middle Bronze Age III, fitting with the current chronological understanding of the beginning of the production of monochrome black ware in northern Iran [14]. The other four dates (TKA-28177, TAK-28178, TAK-28179, and TAK-28183) demonstrate the existence of the late Iron Age burials at Ghalekuti. Those for our IA III agree with previous estimations from the literature based on analogies with other Iron Age settlements [17, 27].

### 3.2 Petrography analyses of pottery sherds

**Fig 4:**
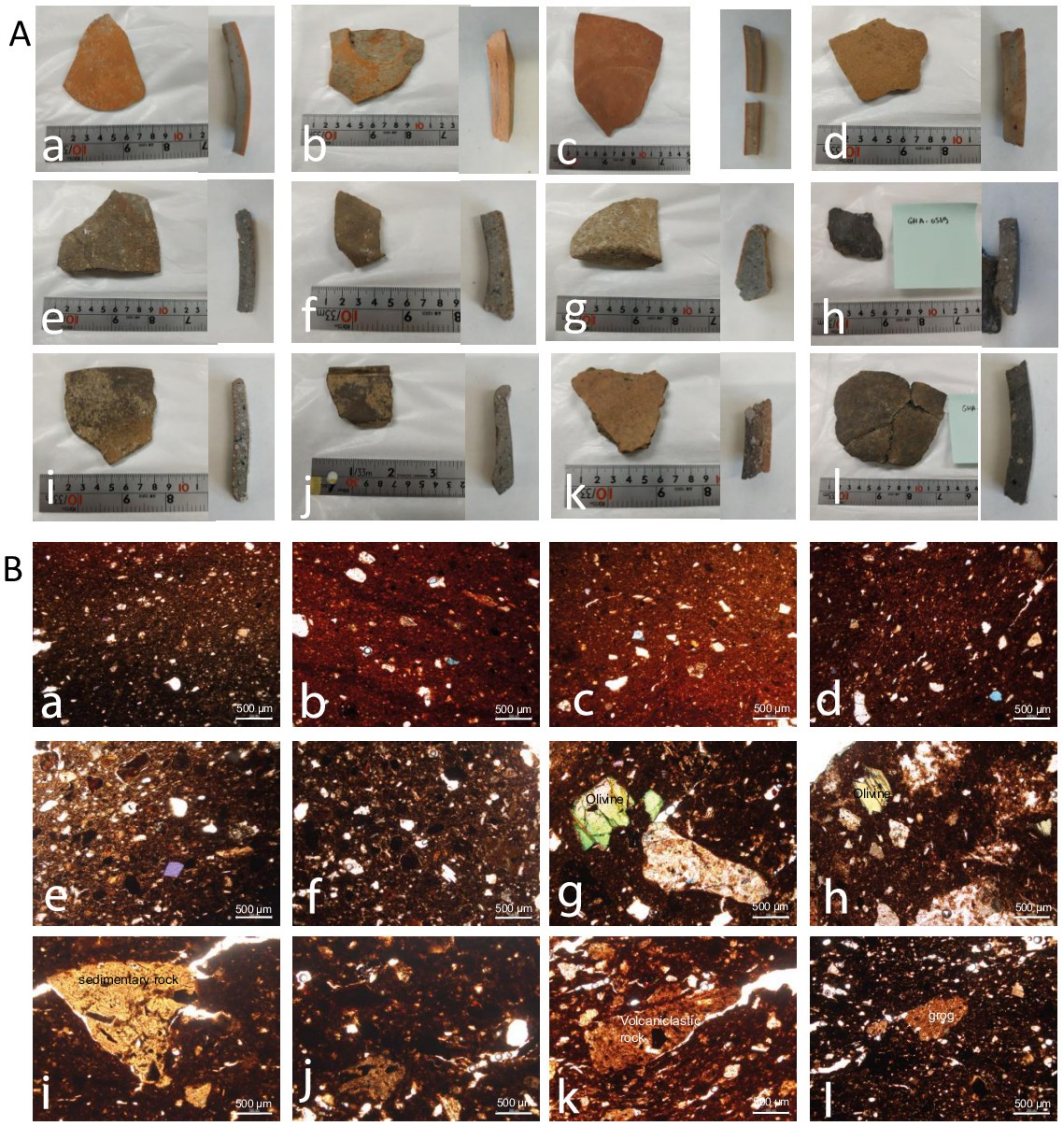
Iron Age ceramic studied as thin-section A. Original sherd and transversal section (macroscopic scale) and B. Thin-sections of the same potsherds observed under XP light from Ghalekuti both sorted by fabric classification upon thin-section observations. Fabric (i): a GHA-0582, b GHA-0583, c GHA-0584, d GHA-0586. Fabric (ii): e GHA-0588, f GHA-0590. Fabric (iii): g GHA-0587, h GHA-0589. Fabric (iv): j GHA-0569, k GHA-0570. Fabric (v): k GHA-0585. Fabric (vi): l GHA-0581.

Five ceramics from BA/IA I and seven from IA III, covering a range of clay colours, were studied as thin sections to obtain information on their manufacture (Fig 4A). This consists of the study of clay variations in composition, examines if these are related to the technological differences observed in macroscopic studies [3], and gains insights into performance characteristics related to the pottery function. The thin sections exhibit common features for all the ceramics: they have fabrics with a mineral inclusion consisting of common inclusions of opaque minerals, and variable inclusions of quartz, olivine, pyroxene, and limestone. No vegetal temper was evidenced in the selection. Nonetheless, six classes of fabric (i to vi) were determined from the studied samples (Table 3; Fig 4B).

**Table 3:** Fabric composition of ceramic vessels studied as vertical thin-section. The percentages and frequency of inclusions followed the ranges defined in Quinn [2,4].

Fabric name	Fabric composition description	POTIRAN# (context, phase)
(i) Iron-rich fabric	**Inclusions** = 15–25% well-sorted inclusions of sharp angular shape minerals. Common inclusions of opaque mineral (0,05–0,2mm), Few/common inclusions of olivine (0,05–0,2mm), Few inclusions of quartz Very rare inclusions of biotite, chlorite and pyroxene. **Matrix =** 73–83% non-calcareous matrix. Red-clay in PPL and XP, optically inactive. Colour gradient between red and grey (macroscopic observation). **Voids** = 2% vesicle, channels and vughs of small sizes.	GHA-0582 (AIII, IA III), Fig 4a
		GHA-0583 (CI, IA III), Fig 4b
		GHA-0584 (BIII, IA III), Fig 4c
		GHA-0586 (IIIB, IA III), Fig 4d
(ii) Opaque mineral-included medium fabric	**Inclusions** = 30% of moderately well-sorted mineral inclusions Frequent opaque minerals, including opaque iron and clay pellet (0,1–0,5mm) Few olivine and quartz (0,05–0,5mm). Very rare limestone (1mm) and pyroxene. **Matrix =** 68% non-calcareous clay matrix of uniform brown colour in PPL and XP, optically inactive. **Voids** = 2% vesicles and vugh of small sizes	GHA-0588 (BIII, IA III), Fig 4e
		GHA-0590 (CI, IA III), Fig 4f
(iii) Ultrabasic rock-included coarse fabric	**Inclusions** = 30% of moderately/poorly sorted mineral inclusions. Frequent inclusions of opaque mineral, including opaque iron (0,1–0,5mm), Few to rare olivine and quartz (0,05–0,5mm). Variable abundance of coarse/medium inclusions of ultrabasic rock fragments (0,2-1mm) and limestone (0,5-1mm). **Matrix =** 58–62% non-calcareous clay matrix of uniform dark brown colour in PPL and XP, optically inactive. **Voids** = 5–8% planar voids, vesicles and vughs. Irregular	GHA-0587 (BIII, IA I), Fig 4g
		GHA-0589 (AIII, IA I), Fig 4h
(iv) Sedimentary rocks with opaque mineral-included coarse fabric	**Inclusions** = 25–35% of moderately/poorly sorted mineral inclusions Frequent inclusions of opaque mineral (0,1–0,5mm), Few to rare olivine and quartz (0,05–0,5mm). Variable abundance of coarse/medium inclusions of sedimentary rock fragments **Matrix =** 62–73% non-calcareous clay matrix of uniform dark brown colour in PPL and XP, optically inactive. **Voids** = 2–3% planar voids, channels, vesicles and vughs of small sizes.	GHA-0569 (AIII, IA I), Fig 4i
		GHA-0570 (AIII, IA I), Fig 4j
(v) Volcaniclastic rock-included coarse fabric	**Inclusions** = 30% of very poorly sorted mineral inclusions Dominance of volcaniclastic rock fragments (possibly altered tuff, 0,2-3mm), Common opaque mineral inclusion (0,1mm) Few olivine and quartz (0,1–0,2mm). **Matrix =** 60–65% non-calcareous clay matrix of uniform dark brown colour in PPL and XP, optically inactive **Voids** = 5–10% planar voids, channels, vesicles and large vughs. Long channels around coarse inclusions parallel to the section, that may have formed during drying.	GHA-0585 (B, IA III), Fig 4k
(vi) Grog and limestone-included coarse fabric	**Inclusions** = 25–30% of moderately/poorly sorted mineral inclusions. Frequent inclusions of opaque mineral (0,1-1mm) Few to rare olivine and quartz (0,05–0,5mm). Variable abundance of coarse/medium inclusions of grogs (0,2-1mm) and limestone (0,5-1mm). **Matrix =** 60–65% non-calcareous clay matrix of uniform dark brown colour in PPL and XP, optically inactive. **Voids** = 5% vesicles, vughs, and channels. Large voids close to the surface alongside inclusions.	GHA-0581 (AIII, IA I), Fig 4l

The first fabric (i) is an iron-rich red clay matrix with common, well-sorted inclusions of opaque iron minerals and olivine. It was only identified for IA III potsherds, the phase when the red/orange clay appeared. Macroscopic observations of thin sections revealed colour gradients from red to grey, suggesting variations in the iron oxidation state during firing [24]. The fabric (ii) has a brown clay matrix with abundant opaque minerals, few inclusions of olivine, and very rare inclusions of limestone. These are from two IA III potsherds. This fabric presents similarity to fabric (i), but the former is different from the latter in that the inclusions are of medium-small sizes in a brown-grey matrix.

The fabric (iii) consists of variable coarse and medium inclusions of igneous rock fragments, common opaque mineral inclusions, and a few inclusions of olivine and limestone. This fabric type was only identified in BA/IA I potsherds. Fabric (iv) is comprised of angular coarse fractions of sedimentary rock fragments with opaque mineral inclusion. This fabric group is also unique to BA/IA I ceramic samples. The fabric (v) was identified in only one potsherd of IA III. It consists of coarse volcaniclastic rock fragments (possibly altered tuff)-tempered brown clay matrix alongside common opaque mineral and few olivine inclusions. These rock fragments are of various sizes and present visible opaque mineral inclusions. Finally, the fabric (vi) consists of variable coarse and medium inclusions of grogs, limestones, or common opaque mineral inclusions, and few olivine inclusions. This fabric type was only identified in BA/IA I potsherds.

The angular shape and relatively large grain size inclusions present in fabric (iii) to (vi) suggest the intentional tempering of coarse grains to the ceramic vessels. These contrast with fabric (i) and (ii) that likely showed naturally occurring inclusions only.

Interestingly, all the variations in colours around the brown (e.g. dark-brown, grey-brown…) and the red (orange, red-brown…) visible at the macroscopic scale (Fig 4A) do not show significant diversity of the nature of small sizes mineral inclusions as thin-section. This suggests that colour differences in the ceramic clay, especially the red specimens are more likely related to firing temperatures and oxidising atmosphere rather than variable clay sources other than the brown and red clay. The main difference in composition is visible between BA/IA I and IA III pottery. The latter phase shows red-based (i) and brown based (ii) clay specimens with medium/fine well-sorted inclusions (with one exception: the fabric v). This thin-section study suggests, to some extent, a continuity in ceramic production with the same raw materials being employed that could suggest a standardised production of ceramics. The red clay-based specimens introduce a more refined pottery production in Iron Age III with mineral inclusions of the same nature as the preceding phases.

In addition, some fabric types show the intentional addition of coarse minerals/ rock fragments/ grogs (fabrics iii–vi) as temper. These coarse-mineral-tempered fabrics may have enhanced the heating effectiveness of ceramic vessels, which suggests one of their functions might be cooking [4]. In contrast, the finer fabric (i) in the red clay might have been less used for cooking due to its physical properties. Serving and transport of goods was possibly the main function.

### 3.3 Lipid residue analyses of pottery vessels

A total of 40 vessels, covering a range of ceramic typologies from the BA/IA I, IA II, and IA III periods, were studied for their lipid residue analyses (Fig 2, Table 1). All the specimens displayed fatty acids (FAs) with a mean fatty acid concentration of 93 μg of lipids per gram of sherd, with a maximum concentration of 500 μg of FA per gram of sherd. From the pottery vessel selection, some display obvious petroleum contamination, probably from the storage and use of plastic bags; some display contamination with insecticides, likely arising from curation practices, and some contamination with polyethylene glycol (PEG) resin, likely arising from restoration work. In most cases, these contaminations occur in trace amounts distinguishable from the archaeological lipid signal, allowing lipid quantification and analyses with GC-IRMS and identification of lipid sources of archaeological origin. Five samples had significant amounts of PEG contamination; thus, δ^13^C values on individual FAs were judged unreliable and are not reported in these specific cases.

**Fig 5:**
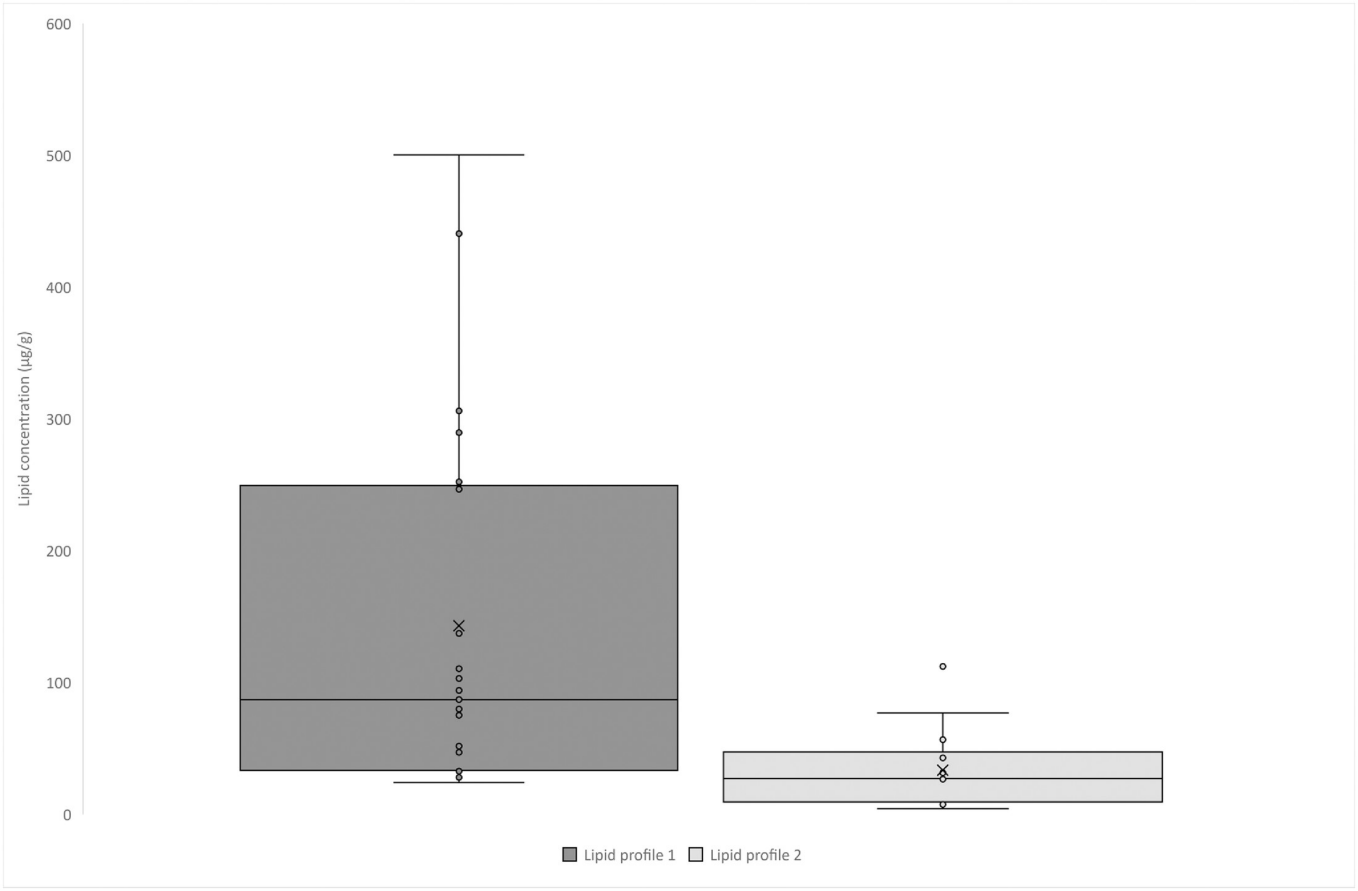
Box and whisker plot showing total C_16:0_ and C_18:0_ concentration based on the lipid profile.

**Fig 6:**
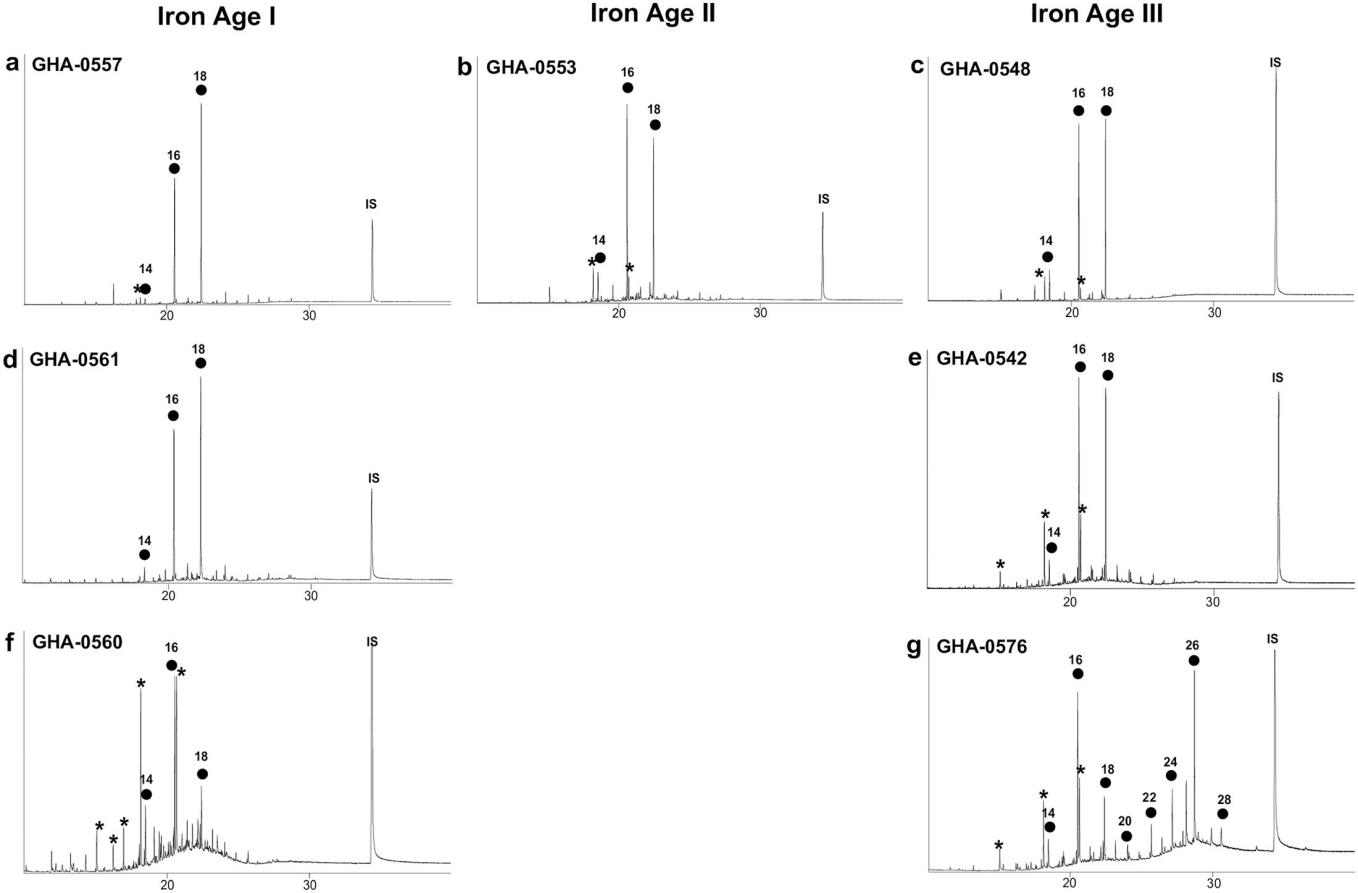
Chromatograms of lipid profile 1 typical of degraded animal fats identified as **a,b,c** ruminant dairy fats, and **d,e** ruminant adipose fats and chromatogram of lipid profile 2 **f,g** suggesting plant oil residues. Dots are *n*-alkanoic acids, numbers their chain length, *denotes modern contaminants (plasticizers and pesticides) and IS the internal standard (*n*-tetratriacontane).

**Fig 7:**
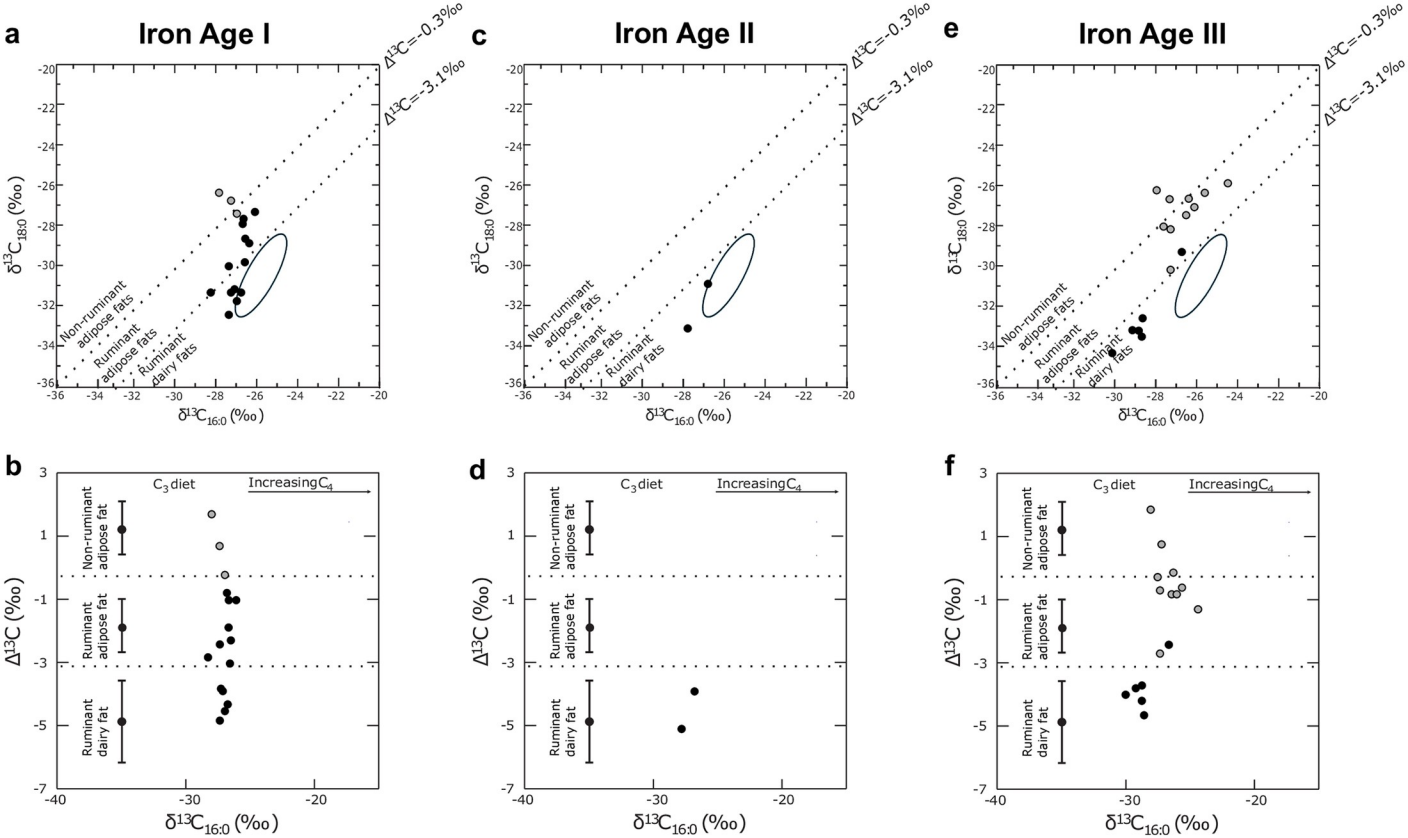
a, c, e. δ^13^C_18:0_ values plotted against the δ^13^C_16:0_ values of the BA/IA I, II, and III ceramic vessels, respectively. b,d,f Δ^13^C (= δ^13^C_18:0_- δ^13^C_16:0_) values plotted against the δ^13^C_16:0_ values for the same ceramics. Blacks dots are TLEs with lipid profile 1 and grey dots TLEs with lipid profile 2. Ellipses denote modern reference range of dairy products from Iran [27], and ranges denotes means values and standards deviation of reference animal fats worldwide [28–31].

Aside from obvious modern contamination, two main patterns emerged in the lipid profiles of the vessels. Most Total Lipid Extracts (TLEs) are dominated by C_16:0_ and C_18:0_ FAs, with the C_14:0_ FA in much less abundance, with an average total FA concentration of 137 μg of FA per gram of sherd (Fig 5). This lipid profile is characteristic of degraded animal fats (*n* = 22, Fig 6a–6e). These TLEs have δ^13^C_16:0_ and δ^13^C_18:0_ values characteristic either of ruminant dairy fats (*n* = 12) or ruminant adipose fats (*n* = 9; Fig 7). The ruminant dairy fats either fit nicely within the modern reference range from Iran [28] or are more depleted, suggesting animal grazing on various pastures, with one having an extremely C_3_ signal comparable to European temperate environments [29]. The ruminant adipose and dairy products can originate from domesticated herds of caprine or cattle for which remains were identified at the site. It should be noted that the partial faunal identification published by Egami *et al*. [8] has been reassessed. It ruled out the presence of gazelle, as the remains previously identified as such were, in reality, caprine remains (S. Arai, personal communication).

The second lipid profile corresponds to TLEs dominated by the C_16:0_ FA, followed by the C_18:0_ and C_14:0_ FAs in about similar relative abundances (*n* = 13, Fig 6f and 6g). This profile has a significantly lower lipid concentration than profile 1, with an average of 33 μg of FA per gram of sherd. This lipid signal might suggest the presence of plant lipids in the vessels. Plant oils are usually dominated by the C_16:0_ over the C_18:0_ FA, and some plants from tropical environments display a significant abundance of short-chain fatty acids (C_12:0_, C_14:0_) in their lipid distribution. Supporting this hypothesis of plant exploitation in pottery, two TLEs display, in addition, long-chain fatty acids with an even over odd number chain length dominance, maximising on the C_26:0_ FA (Fig 6g), which are common from plant leaf or epicuticular waxes. This lipid distribution is common in plant leaves or epicuticular waxes [32, 33]. No long-chain *n*-alkanes were detected in the vessels, which could suggest plant oil residue rather than plant wax residues. The identification of a more precise origin of the plant oil exploited is beyond reach. All these residues display δ^13^C_16:0_ and δ^13^C_18:0_ with average values of -27.1‰ which corresponds to C_3_ plants. These plots in the range of non-ruminant adipose (*n* = 7) or ruminant adipose fats (*n* = 6) in the animal fats proxy values (Fig 7).

There is also the possibility of the mixing of animal fats with plant lipids in some of these vessels. This point is suggested by (i) the presence of long-chain FA in small abundances in TLEs of profile 1, (ii) the TLEs of lipid profile 2 and δ^13^C_16:0_ and δ^13^C_18:0_ values plotting in the range of ruminant adipose fats close to the border with dairy fats region. No other lipid profiles were detected in the pottery assemblage studied, suggesting a rather limited origin of goods used in funerary vessels.

### 3.4 Temporal relation between typology and content

#### 3.4.1 Bronze Age/Iron Age I assemblage

During the BA/IA I period, most of the residues identified in funerary ceramics are characteristic of degraded animal fats with ruminant dairy fats (*n* = 5) or ruminant adipose fats (*n* = 8).

The dairy fat residues were discovered in a bowl with a foot, a straight bowl, a bowl with a spout, a jar with two handles and feet, and a small ceramic in the shape of a goblet (Fig 2d, 2c, 2f, 2h and 2t). This range of vessel types and sizes suggests that not a single pottery type was used for the processing of dairy products. Dairy products could have been employed in diverse forms (e.g., milk, butter, cream, etc.). The bowl with the spout implies, however, the use of dairy under a liquid state such as fresh, fermented milk or a milk-based product.

The TLEs with a dominance of ruminant adipose fats were discovered in a short neck jar with two handles, a tall neck, and bowls of various sizes (Fig 2b, 2d, 2h, 2k, 2u and 2v). Some have δ^13^C_16:0_ and δ^13^C_18:0_ close to the border with the dairy region, which could suggest a mixing of both dairy and adipose products with a dominance of adipose fats. The tall neck jar has a typology suggesting that adipose fats would have been in a liquid state due to its narrow neck, which could correspond to products such as tallow, blood, or meat juice preparation from ruminant animals.

Three vessels display lipid profile 2 assigned to vegetable oil residues. These lipid profiles were identified in one very large bowl, one straight bowl with a tubular spout, and a small cup (Fig 2a, 2e and 2s). Here again, the ceramic vessels are of various types and sizes without an apparent relationship between the goods and typology. The straight bowl with a tubular spout suggests some plant/vegetable oil in a liquid state to have been poured. It should be noted that the bowl with a tubular spout and the small cup are unique vessels in the BA/IA I assemblage. These particular vessels could have been created for a purpose related to the use of plant-based products only.

In addition, the thin-section study of BA/IA I pottery suggests intentional tempering with mineral inclusions, which could suggest the search for heat-effective fabric. This could suggest either a domestic use of the vessel before the burial of the deceased or their use in funerary rituals involving the heating of commodities.

#### 3.4.2 Iron Age II assemblage

Only four vessels from IA II were studied due to the small number of IA II materials discovered at Ghalekuti compared to other periods. Two of the pottery vessels contained residues dominated by ruminant dairy fats, while the other two contained obvious contamination with PEG resin. The two vessels with dairy fats correspond to a straight bowl and a jar with two handles (Fig 2c and 2h). These two pottery typologies were found to contain dairy products during the BA/IA I period as well, suggesting some sort of continuity in the processing of ruminant dairy in such vessel types. None of the IA II ceramic vessels were suited for thin section petrography to study their mineral composition.

#### 3.4.3 Iron Age III assemblage

During the IA III period, pottery vessels with lipid profile 1, typical of degraded animal fats residues, are dominated by ruminant dairy fats (*n* = 5) followed by ruminant adipose fats (*n* = 1). Dairy residues were identified in two small spouted bowls with handles, one open bowl, one short neck jar, and a long neck jar with a tubular spout opposing the handle (Fig 2f, 2g, 2j and 2o). The typology of spouted vessels suggests the use of dairy in a liquid state in those vessels to be poured through the spout, thus similar to fresh or fermented milk as seen in the BA/IA I phase. The ruminant adipose fats were recovered in one spouted bowl (Fig 2f). These suggest a liquid state for the adipose fats, such as tallow, blood, or meat juice.

The remainder of the TLEs (*n* = 10) display lipid profile 2, suggesting plant oil residue. The δ^13^C values plot within the range of non-ruminant adipose fats (*n* = 4) or ruminant adipose fats (*n* = 6). All of the vessels are long-neck jars (*n* = 6) or jars with a tubular spout perpendicular to the handle (*n* = 4) (Fig 2m, 2n, 2p, 2q, 2r and 2o). These suggest some uniformity in the use of long neck jars with or without a tubular spout for plant-based products. It should be noted that the lipid concentration of two long neck jars plotting in the ruminant adipose region is weak, and lipid distribution at the edge of lipid profile 2 with some similarity to lipid profile 1 may indicate here the mixing of ruminant adipose or even ruminant dairy and plant lipids.

The IA III phase displays some level of vessel specialisation regarding the use of animal products (adipose and dairy) and botanical products. Ruminant products appear more particularly processed in bowls (spouted or not) and short-neck jars, whereas the botanical products are exclusively recovered in long-neck jars and long-neck jars with a tubular spout. The long-neck jars are almost exclusively made in the red-clay. The thin section study of red-clay vessels and brown-clay vessels (at one exception) of IA III evidence no intentional tempering, meaning these would not have been suited for heating. This could suggest the function of these ceramics to have been more particularly dedicated to funeral practices such as libation rather than domestic use before the burial.

## 4. Discussion

The ceramic typology and manufacture evolve with age, introducing new forms of jars and raw (red) clay material while maintaining certain levels of continuity with the use of brown clay matrix, bowls, and small jars at all phases.

During the BA/IA I period, there appears not to be a direct correspondence between pottery types and the use of animal/botanical products. However, botanical products have been found in two vessels that are unique in the assemblage, suggesting the possibility that these were made specifically for such products. During the IA III period, the appearance of jars with tall necks and tubular spouts in red clay seemed more particularly dedicated to botanical products (reflecting a growing importance in the use of such products). The use of adipose products appears to decrease in abundance from the BA/IA I to IA III period. We do not exclude the possibility that the pottery with lipid profile 2 at the edge of lipid profile 1 could be the result of the mixing of ruminant products and plant oil. This picture could also correspond to a bias in sampling, as most IA III ceramics are from the same grave (C-I).

One noticeable finding is that dairy products were used during the Iron Age at the site supposedly for rituals. They were used as fresh or fermented milk or other milk-based mixtures in several vessels with a spouted typology. It should be noted that dairy products were mainly recovered in bowls, spouted bowls, and short-neck jars with or without handles. These vessel typologies seem to serve a similar function across the IA periods, mainly for animal products. While the dairy residues in BA/IA I and II pottery fit well within the modern reference range of dairy available for Iran (Fig 7b and 7d) [28], the dairy residue in Iron Age III pottery has a more depleted signal (Fig 7f), suggesting feeding on much more C_3_ pastures [29] et al., 2003). This implies that the ruminant herds in BA/IA I and IA III had access to diverse grazing pastures. It seems rather unlikely that the pasture changed quickly in the region. Considering most of the IA III ceramics are from the same grave, the possibility that only one animal (e.g., cattle), which came from another region or had a particular diet, provided all the milk used in the ceramics from the grave exists.

ORA revealed that all the funerary vessels were used before their deposition in the graves. However, the lack of known domestic occupation in the region prevents comparison of these funerary typologies with domestic typologies to ascertain if these were used in rituals or during domestic purposes in the lifetime of the deceased. In this regard, the pottery assemblage from the upper layer of Tomb B-III deserves particular attention because it could have been derived from domestic contexts [10]. Yet, the sample size for B-III is too small to make a reliable comparison in the pottery uses between the domestic and funerary contexts, not more than stating that long-neck jars recovered from both contexts similarly exhibit traces focusing on the use related to vegetable oil (Table 1). Understanding the diagnostic use of pottery for funeral rituals will be promoted with data from its comparisons with the pottery use patterns at domestic settlements [34], which have not been studied from this point of view.

Nonetheless, the burial goods at Iron Age graves in central and North Iran (for instance at Sialk, Kashan, Iran) characteristically contain highly decorated pottery vessels that can hardly be considered for daily use [17, 27, 35]. This fact has formed a common hypothesis that the grave goods pottery would have had a purpose and use in funerary practices. A large number of vessels with tall necks and spouts, based on their specific typology, imply the pouring of liquid commodities. Therefore, libation, a ritual involving the pouring of liquids or grains, may have played a significant role in Iron Age funerary practices. This finding encourages a further study of pottery vessels of the Iron Age of Iran with reference to the historical texts (e.g. [36–38]).

## 5. Conclusion

Archaeological records from the large necropolis at Dailaman serve as a valuable source of information to understand the funerary practice of the Iron Age in North Iran. While the current research on pottery vessels in funerary practices has focused on typological analysis, the present study applies ORA and thin-section petrographic analysis to the pottery vessels in the grave goods for the first time. ORA and sampling in strategic places of the vessels (e.g., spouts) revealed that ceramic vessels from funerary contexts at Ghalekuti I and II were actively used for goods of animal and botanical origins before their deposition in the graves. This raises a question as to the notion that lavish grave goods were prepared for purely ritual purposes without actual use. Furthermore, the pottery typology revealed a specialisation of jars with tubular spouts and long necks, particularly employed for botanical products (e.g., plant oil), whereas bowls and short-neck jars were more used for animal products, including dairy products. A large part of the goods employed were likely used under a liquid state and potentially used for some sort of libation practices.

The new approach thus revealed previously unknown aspects of pottery use among the Ghalekuti communities. The study of other funerary pottery assemblages, as well as domestic assemblages with ORA, would help in obtaining a more comprehensive picture of the domestic and ritual use of pottery vessels across the Iranian Plateau during the Iron Age. Furthermore, a significant number of other materials are deposited in the graves (weapons, jewellery, etc.). Their study would complement the picture of funerary practices we started to obtain through analysis of the pottery assemblages.
